# Pneumomediastinum and pneumopericardium 11 days after Whipple procedure. A case report and review if the literature

**DOI:** 10.1016/j.ijscr.2020.04.012

**Published:** 2020-05-08

**Authors:** Gavriella Zoi Vrakopoulou, Victoria Michalopoulou, Christina-Evaggelia Kormentza, Maria Matiatou, K. George Zografos, Konstantinos G. Toutouzas

**Affiliations:** 1st Propaedeutic Surgical Department, Hippocratio General Hospital, Medical School, National and Kapodistrian University of Athens, Greece

**Keywords:** Spontaneous pneumomediastinum, Spontaneous pneumopericardium, Postoperative pneumopericardium, Pneumomediastinum after Whipple, Late complication after Whipple

## Abstract

**Introduction:**

Spontaneous pneumomediastinum (SPM) is a rare condition classified as free air in the mediastinum in the absence of any precipitating cause. This is the first time that a synchronous presence of pneumopericardium is described. To date, there are no clear guidelines for diagnosis and treatment.

**Presentation of a case:**

A 34-year old Caucasian male patient presented to our institution with a recently diagnosed pancreatic adenocarcinoma. He underwent an uneventful pancreaticoduodenectomy (Whipple procedure). Preoperative and intraoperative chest X-Ray after a central line placement were normal. The postoperative course was uneventful, but few hours before his discharge he presented an acute tachycardia and tachypnea with hypocapnia and a transient loss of consciousness. The full-body CT scan revealed a pneumomediastinum and pneumopericardium without any findings of anastomotic leak or other pathology from the abdomen. A meticulous review of the literature was conducted about the pathophysiology, treatment options and outcomes of pneumomediastinum after a surgical procedure.

**Discussion:**

This is the first study presenting the case of spontaneous pneumomediastinum with a synchronous pneumopericardium in the literature as a late complication of Whipple procedure. The applied diagnostic algorithm and conservative treatment are presented to extend our limited knowledge about this rare medical entity.

**Conclusion:**

Awareness of these medical entities is important for the adequate management and optimal outcome of patients presenting a spontaneous pneumomediastinum. As such, all cases, treatment decisions and outcomes should be reported.

## Highlights

• Spontaneous Pneumomediastinum is a rare disease.
• Combination with Pneumopericardium has not been reported yet.
• There are no clear guidelines for diagnosis and treatment.
• Conservative treatment with close monitoring is most of the times sufficient.
• In surgical patients an upgraded diagnostic and treatment plan could be necessary.

## 1 Introduction

Pneumomediastinum was initially described by Luennec in 1819 and later by Hamman in 1939 [1]. Spontaneous pneumomediastinum (SPM) is defined as the presence of air in the mediastinum that is not associated with thoracic trauma, respiratory conditions, gastrointestinal or respiratory diagnostic procedures, mechanical ventilation or thoracic surgery [2]. Pathophysiology includes alveolar rupture and transport of air along sheaths of pulmonic blood vessels from alveoli to mediastinum (Macklin effect) [3]. Incidence is higher in young adult men between the ages of 20 and 40 years old [4,5], although some authors report a similar incidence in men and women [2,3]. It is attributed to activities that increase alveolar pressure such as: intense coughing, crying, vomiting and constipation.

There are no clear guidelines for SPM diagnosis and treatment [[6], [7], [8]]. The present manuscript aims to highlight an extremely rare case of SPM with synchronous pneumopericardium and to underline the importance of a prompt diagnosis in a short time frame, and all the necessary examinations for the successful treatment of this medical entity in a postoperative patient.

The patient was managed in an academic practice setting. This work is reported according to the SCARE criteria [9].

## 2 Case report

A 34- year- old Caucasian male patient with a Body Mass Index (BMI) of 32 kg/m^2^, a personal history of type II Diabetes Mellitus and myocardial infarction was treated with a pancreaticoduodenectomy (Whipple procedure) for pancreatic adenocarcinoma. According to the preoperative assessment protocols of our department, a preoperative chest – X – ray was performed and no pathological findings were found. Intraoperatively, a central jugular line was placed and a control chest X-Ray was performed to confirm the right position of the catheter and to exclude complications such as the existence of pneumothorax or pneumomediastinum. The postoperative course was uneventful and the patient started on 5th postoperative (p.o.) day with a liquid diet.

On 11th p.o. day patient developed tachypnea (up to 40 breaths per minute) and tachycardia (120 beats per minute) followed by a transient loss of consciousness. Blood pressure was 90/60 mmHg. After oxygen supplement, the level of consciousness quickly improved and the Glasgow coma scale was estimated at 14/15. Physical examination revealed no signs of subcutaneous emphysema. Hence, no auscultatory abnormalities were detected, including Hamman’s sign. Acute myocardial infarction was ruled out.

**Fig. 1:**
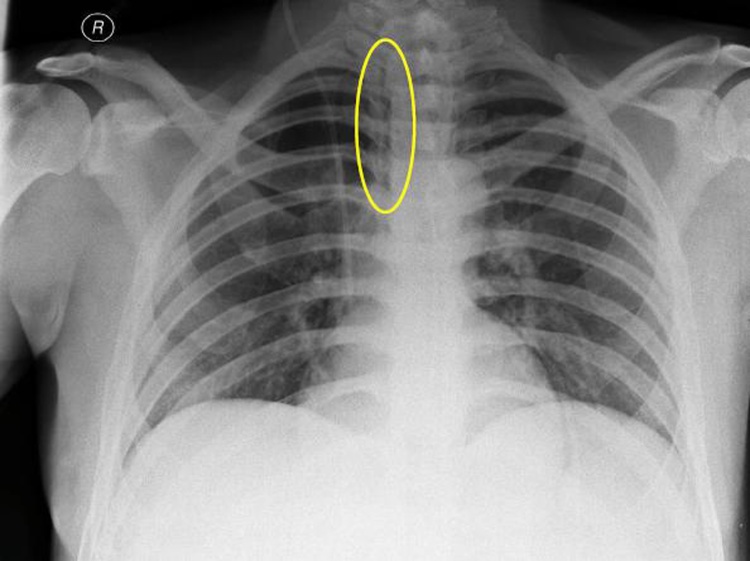
Chest X-Ray. Small amount of air in the mediastinum next to the upper third of the trachea indicating pneumomediastinum.

**Fig. 2:**
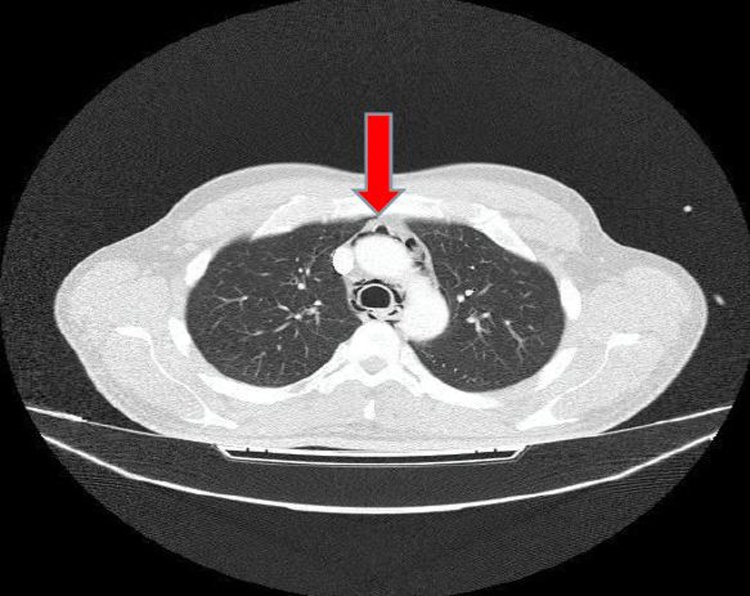
Chest-CT revealing presence of air in the mediastinum.

**Fig. 3:**
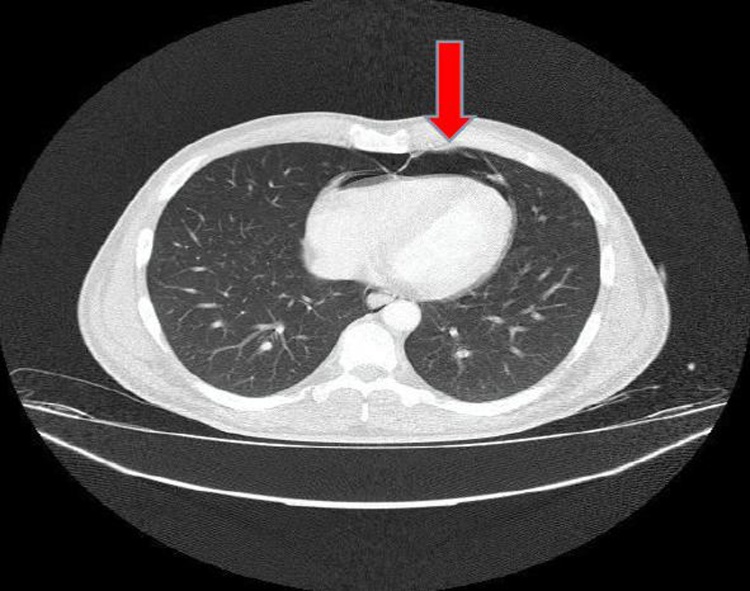
Pneumopericardium on Chest-CT.

The patient was immediately admitted to the High Dependency Unit for further monitoring and underwent a full-body Computed Tomography (CT) which revealed a pneumomediastinum and pneumopericardium (Fig. 1, Fig. 2, Fig. 3), without any signs of pulmonary embolism, pneumothorax, visceral perforation or anastomotic dehiscence in the abdomen. He remained stable, with a nil per os diet and was further assessed by cardiothoracic surgeons to exclude mediastinitis or bronchial rupture. Prophylactic antibiotic treatment was initiated and some examinations were performed for differential diagnosis, such as bronchoscopy, esophagram, and esophagoscopy, but no additional findings, including esophageal and tracheobronchial rupture, were detected.

**Fig. 4:**
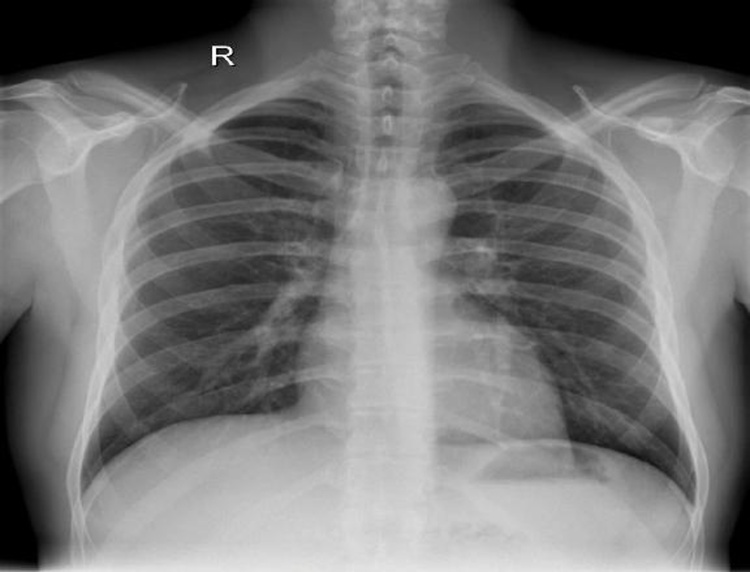
Chest X-Ray without pathological findings 3 months later.

After 24 h, the patient had a control CT scan of the thorax which revealed no alterations from the previous one. His clinical condition gradually improved and the patient could upgrade to a liquid diet. After 72 h of monitoring at the High Dependency Unit, the patient was transferred to the surgical clinic and was discharged from the hospital 10 days later. The last thorax-CT before his discharge showed minimal signs of pneumopericardium and pneumomediastinum. The follow-up chest X-ray, three months later, showed no signs of pneumomediastinum or pneumopericardium (Fig. 4).

## 3 Discussion

Spontaneous pneumomediastinum (SPM) is a rare condition characterized by free air in the mediastinum in the absence of any precipitating cause, with nonspecific signs and symptoms [10]. The combination of SPM with a pneumopericardium is extremely rare and this the first case in the literature reporting a combined presence of these two entities as a late complication of a Whipple procedure.

The exact mechanism of SPM is mostly related to activities that increase the alveolar pressure such as intense coughing, crying, vomiting, and constipation. After a detailed examination of patient’s postoperative course, there was no report of such symptoms or of intense coughing or struggling during respiratory exercises or meal times that could explain SPM and pneumopericardium at this young patient.

After reviewing the literature, we found only few cases describing this medical situation after a laparotomy [11,12]. For these cases, the remaining abdominal air seems to translocate towards the mediastinum resulting in a pneumomediastinum 10 days after a kidney transplantation, but none of them reports the existence of a synchronous pneumopericardium.

There are no clear guidelines for SPM diagnosis and treatment. In a retrospective study of Takada et al. [6], authors claimed that SPM could be a self-limited disease with mild complications and recurrence. It’s management, based on the clinical course, includes a close monitoring without the addition of prophylactic antibiotic therapy, limitation of oral intake or additional examinations. Furthermore, chest CT or esophagram may be added only in suboptimal cases. However, in this study none of the cases was a surgical one and therefore the proposed algorithm for management of SPM was simplified and maybe not sufficient for surgical patients.

For SPM cases reported after kidney transplantations, E.S. Kerns [11] illustrates that creation of a potential space in the abdominal cavity can be associated with the development of SPM, while A. Kis [12] assumes that alveolar rupture, caused by increased intrapulmonary pressure during intraoperative ventilation, was responsible for the case reported by him.

Hence, in a retrospective comparative analysis of 47 SPM cases Caceres M. et al. [5] report that, similar to our case, in 21% of patients no apparent triggering event was noted. Chest radiograph was diagnostic in 69% and computed tomography was required in 31% to establish the diagnosis. In this study, esophagram, esophagoscopy, and bronchoscopy were performed on an individual basis and were invariably negative.

## 4 Conclusion

Spontaneous pneumomediastinum is a benign situation with excellent prognosis, if uncomplicated. Diagnosis is confirmed with a chest X-ray and/or chest computed tomography and a conservative treatment with nil p.o. diet and close monitoring is usually sufficient. The rarity of this medical entity and the lack of specific guidelines for SPM diagnosis and treatment, makes the report of this rare case of spontaneous synchronous pneumomediastinum and pneumopericardium interesting in terms of the applied diagnostic algorithm and treatment.

## Declaration of Competing Interest

Authors have nothing to declare.

## Sources of funding

This research did not receive any specific grant from funding agencies in the public, commercial, or not-for-profit sectors.

## Ethical approval

No ethical approval was required for this case report.

## Consent

A written and signed consent to publish this case report has been obtained.

## Author contribution

Vrakopoulou G.: contributed to the study concept and design, data collection, data analysis and interpretation, and wrote the paper.

Michalopoulou V.: contributed to the study design, data analysis and interpretation, and wrote the paper.

Kormentza C.: contributed to the study concept and design, data collection, and editing of the paper.

Matiatou M.: contributed to the study concept and design, data collection and interpretation, and editing of the paper.

Zografos G.: contributed to the study concept, data interpretation, and editing the paper.

Toutuozas K.: contributed to the study concept and design, data analysis and interpretation, and editing the paper.

## Registration of research studies

NA.

## Guarantor

NA.

## Provenance and peer review

Not commissioned, externally peer-reviewed.
